# Association of the Dietary Inflammatory Index with bone mineral density and osteoporosis among adults with type 2 diabetes: A cross-sectional NHANES study

**DOI:** 10.1097/MD.0000000000050645

**Published:** 2026-09-18

**Authors:** Menglin Xu, Qiqi Tang, Shaocong Li

**Affiliations:** a College of Traditional Chinese Medicine, Chongqing Medical University, Chongqing, China; b Guangzhou University of Traditional Chinese Medicine, Guangzhou, China; c Preventive Treatment Center, Shenzhen Bao’an Authentic TCM Therapy Hospital, Shenzhen, China; d School of Clinical Medicine, Chengdu University of Traditional Chinese Medicine, Chengdu, China.

**Keywords:** bone mineral density, Dietary Inflammatory Index, NHANES, osteoporosis, sex differences, type 2 diabetes

## Abstract

Dietary inflammatory load, quantified using the Dietary Inflammatory Index (DII), reflects pro-inflammatory dietary patterns that may contribute to impaired bone health. However, population-based evidence regarding the association between dietary inflammatory potential and bone mineral density (BMD) among adults with type 2 diabetes (T2D) remains limited, particularly regarding potential sex-specific differences. This cross-sectional study assessed the association between the DII and low BMD (osteopenia or osteoporosis) in adults with T2D. Data from the 2007 to 2010, 2013 to 2014, and 2017 to 2018 cycles of the National Health and Nutrition Examination Survey were analyzed. DII scores were computed from 22 dietary components; participants lacking BMD or DII data were excluded. Low BMD was defined from femoral neck and lumbar spine BMD, with osteoporosis and osteopenia both classified into a single category (“low BMD”). Weighted logistic regression was used to examine the relationship between DII score and low BMD overall and according to sex, adjusted for age, race/ethnicity, body mass index, socioeconomic variables, and clinical confounders. Subgroup analyses, interaction tests, and sex-stratified correlation analyses were performed for consistency. Data from 1768 adults with T2D, of whom 798 had low BMD, were included. Higher DII scores were significantly associated with an increased risk for low BMD in women (femoral neck: odds ratio = 2.575, 95% confidence interval = 1.484–4.468, *P* = .001; lumbar spine: odds ratio = 1.864, 95% confidence interval = 1.111–3.129, *P* = .018), with no significant association observed in men. A significant interaction according to sex was detected (*P* for interaction = .02); however, no interactions were found for age or body mass index. Findings suggested that an elevated DII is independently associated with low BMD in women with T2D. Clinically, sex-specific, anti-inflammatory dietary strategies may help mitigate the risk for low BMD in this population.

## 1. Introduction

Bone mineral density (BMD) refers to the mineral content per unit area of bone and is used clinically to gauge skeletal development and bone mineral status.^[1]^ Osteoporosis (OP) is a systemic skeletal disorder characterized by reduced BMD and deterioration of bone microarchitecture, leading to a markedly increased risk for fracture that constitutes a global public health concern.^[2]^ Globally, an estimated 536.6 million adults aged 20 to 79 years were living with diabetes in 2021, and this number is projected to increase to 783.2 million by 2045.^[3]^ In the United States, data from the National Health and Nutrition Examination Survey (NHANES) indicated that 10.3% of adults aged 50 years or older had OP and 43.9% had low bone mass at the femoral neck or lumbar spine.^[4]^ Diabetes is a core component of metabolic syndrome and is closely associated with the development of OP. Evidence indicates that chronic hyperglycemia, insulin resistance, and oxidative stress in diabetes disrupt bone metabolism, thereby accelerating bone loss and increasing the incidence of the condition.^[5,6]^ With population aging and shifts in lifestyle, the prevalence of diabetes complicated by OP has increased annually. However, the specific etiologies and comorbidity mechanisms remain incompletely defined, thus limiting current prevention and treatment strategies.^[7]^

Chronic low-grade inflammation underlies the comorbidities of diabetes and OP. Pro-inflammatory cytokines, particularly tumor necrosis factor-α, can interfere with insulin receptor signaling and thereby promote peripheral insulin resistance.^[8]^ In pancreatic islets, glucose-induced interleukin-1β (IL-1β) expression and sustained inflammatory signaling may contribute to β-cell dysfunction in individuals with type 2 diabetes.^[9]^ These mechanisms provide a biological rationale for investigating whether the inflammatory potential of diet is associated with bone health in individuals with type 2 diabetes. Hyperglycemia activates pro-inflammatory signaling pathways (e.g., NF-κB), inducing the release of cytokines such as interleukin (IL)-6 and tumor necrosis factor-α, which suppress osteoblast differentiation while enhancing osteoclast activity.^[10,11]^ Recent studies further support the importance of the inflammatory microenvironment in OP, reporting that immunomodulatory approaches reshape osteoimmune signaling and bone remodeling.^[12]^ Dietary interventions, as key lifestyle strategies, can modulate systemic inflammation through pro- or anti-inflammatory food components, potentially mitigating diabetes-associated bone loss. As a key exogenous modulator of inflammation, diet can alter systemic inflammatory status through pro- or anti-inflammatory constituents. Lifestyle strategies, particularly dietary modification, may help mitigate inflammation-related metabolic and skeletal consequences by lowering systemic inflammatory burden. The Dietary Inflammatory Index (DII) is used to quantify the inflammatory potential of dietary components and provides a standardized tool for assessing the putative impact of diet on chronic inflammation.^[13]^ Studies have shown that a higher DII score is significantly associated with elevated levels of inflammatory biomarkers, including C-reactive protein (CRP) and IL-1β.^[14]^ These findings suggest that a diet reflecting high DII scores may contribute to the pathological progression of diabetes and OP by exacerbating inflammatory responses.^[15]^

Although inflammatory mechanisms implicated in diabetes and OP have been partially elucidated, studies specifically addressing the link between dietary inflammatory profiles and OP risk in individuals with diabetes remain scarce. Moreover, evidence from large-scale populations, particularly analyses based on authoritative datasets such as the NHANES, has not yet clarified the predictive value of the DII for diabetes-related OP. As such, the present study used data from the NHANES to examine differences in OP risk across DII strata among adults diagnosed with diabetes to provide a scientific basis for dietary interventions that ameliorate inflammation and reduce the risk for OP. We hypothesized that a higher DII would be associated with a higher risk for OP, with a stronger association observed in women than in men.

## 2. Materials and methods

### 2.1. Study population

Data were drawn from the NHANES, a nationally representative program that collects demographic, socioeconomic, dietary, and health information along with selected medical, dental, and physiological measurements and laboratory results obtained by trained personnel. The NHANES uses a complex multistage probability sampling design, and all survey protocols were approved by the National Center for Health Statistics. Participants provided informed written consent. The present analysis used de-identified public data and was, therefore, exempt from an additional institutional review.

**Figure 1.**
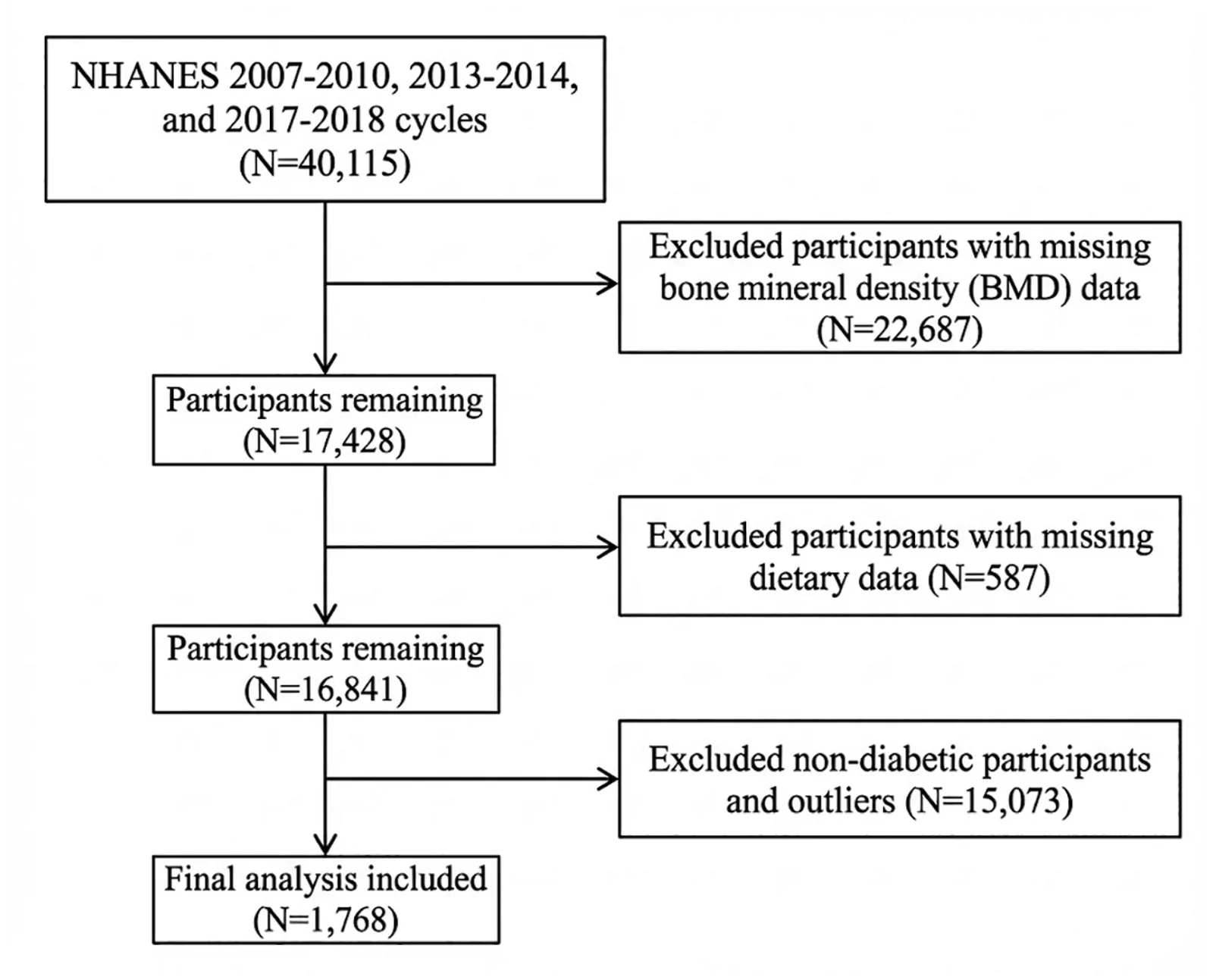
Flowchart of participant inclusion. NHANES = National Health and Nutrition Examination Survey.

The present study included data from adults who participated in NHANES in 2007 to 2010, 2013 to 2014, and 2017 to 2018. The inclusion criterion was physician-diagnosed diabetes mellitus. The exclusion criteria were as follows: missing BMD data at the lumbar spine or femoral neck; missing DII components or implausible DII values (≥20 or ≤−20); and missing sampling weights. The patient selection process is illustrated in Figure 1.

### 2.2. Exposure and covariate assessment

#### 2.2.1. Research design

The following data were extracted from the NHANES database: age, race/ethnicity (Mexican American, non-Hispanic White, non-Hispanic Black, other Hispanic, and other race), household income, body mass index (BMI), glycated hemoglobin A1c (HbA1c), waist circumference, smoking status, alcohol consumption, BMD, and DII. Data were obtained via the NHANES website (https://www.cdc.gov/nchs/nhanes/).

The DII was used as a dietary assessment tool to quantify the overall inflammatory potential of each participant’s diet. Twenty-two dietary components were included: carbohydrate, cholesterol, protein, alcohol, niacin, total fat, vitamin B_6_, vitamin C, zinc, iron, dietary fiber, energy, vitamin B_12_, carotene, caffeine, folate, magnesium, monounsaturated fatty acids, polyunsaturated fatty acids, selenium, vitamin D, and vitamin E. For each component, a DII score (*X*) was calculated by standardizing the individual daily intake against the global reference (*X* = [intake − global mean]/global standard deviation [SD]) and multiplying this standardized value by the published inflammatory effect score for that component. The inflammatory effect scores were derived from previously published studies that developed and validated the DII and quantified the pro- or anti-inflammatory contributions of each component. The total DII for each participant was obtained by summing the 22 component-specific scores.^[13]^ Participants were stratified according to sex, and the DII was separately categorized into tertiles for men and women. For each sex, individuals were assigned to DII model 1 (lowest tertile), DII model 2 (middle tertile), or DII model 3 (highest tertile). The abovementioned indicators were then compared across the 3 DII groups for each sex.

Smoking status was defined using NHANES smoking questionnaire items. Never smokers were those who reported smoking <100 cigarettes in their lifetime (SMQ020 = 2). Ever smokers were those who reported ≥100 cigarettes (SMQ020 = 1). Among ever smokers, current smokers were those who currently smoked cigarettes every day or some days (SMQ040 = 1/2), whereas former smokers were those who did not currently smoke (SMQ040 = 3). Accordingly, occasional smoking (e.g., 1 cigarette per week) would be classified as current smoking if the participant reported smoking “some days.” Alcohol consumption was categorized based on the NHANES Alcohol Use Questionnaire as current drinking versus noncurrent drinking (e.g., any alcohol use in the past 12 months vs none).

#### 2.2.2. OP assessment criteria

According to World Health Organization definitions, OP is diagnosed at a *T*-score ≤−2.5,^[16]^ whereas osteopenia is defined as −2.5 < *T*-score ≤ −1.0.^[17]^ After obtaining the areal BMD, the *T*-score was calculated as follows: *T*-score = (participant BMD − reference mean BMD)/reference SD.^[18]^ In this study, BMD of the lumbar spine and femoral neck were analyzed. OP and osteopenia were combined to comprise the low BMD group, with participants with normal bone mass serving as the reference group. This combined outcome was prespecified to capture clinically meaningful reduced bone mass across the continuum from osteopenia to OP and to improve the statistical power for sex-stratified analyses, given the relatively small number of OP cases analyzed separately.

### 2.3. Statistical analysis

Data were analyzed using R version 4.2.2 (R Foundation for Statistical Computing), SPSS version 27.0 (IBM Corporation), and Stata Release 18 (StataCorp LLC). To ensure national representativeness, all analyses incorporated NHANES sampling weights (strata and primary sampling units). Continuous variables are expressed as mean ± SD, and categorical variables as count and weighted percentage. Between-group differences were assessed using survey-weighted linear regression for continuous variables and survey-weighted chi-squared tests (Rao–Scott) for categorical variables.

Survey-weighted binary logistic regression models were used to examine the association between DII and low BMD, as follows: model 1, unadjusted; model 2, adjusted for age and race/ethnicity; and model 3 was additionally adjusted for household income, waist circumference, BMI, HbA1c, and smoking status. All statistical tests were two-sided, and differences with *P* < .05 were considered to be significant.

## 3. Results

### 3.1. Baseline characteristics

Data from 1768 participants were included, of whom 798 comprised the low BMD group; all summaries and comparisons were survey-weighted. Characteristics of participants with and without low BMD are summarized in Table 1. Compared with the normal BMD group, the low BMD group was older (66.3 ± 11.7 vs 58.6 ± 13.2 years) and included a higher proportion of women (49.5% vs 41.4%). The normal BMD group exhibited higher BMI (32.2 ± 5.9 vs 29.0 ± 5.3 kg/m^2^), slightly higher HbA1c (7.5 ± 1.8% vs 7.2 ± 1.5%), larger waist circumference (109.1 ± 14.0 vs 103.3 ± 13.6 cm), and higher fasting plasma glucose level (9.19 ± 3.91 vs 8.33 ± 3.62 mmol/L), whereas 25-hydroxyvitamin D level was lower in the low-BMD group (57.49 ± 22.75 vs 63.82 ± 24.36 nmol/L). Smoking status, household income, and serum calcium, phosphorus, and uric acid levels did not differ significantly between the groups (Table 1).

**Table 1:** Baseline characteristics comparison (weighted) (*x̅* ± s, n [%]).

Project	Sum (n = 1768)	Normal BMD group (n = 970)	Low BMD group (n = 798)	*P*
Age	62.1 ± 13.1	58.6 ± 13.2	66.3 ± 11.7	<.05
Sex				<.05
Male	964 (54.5)	564 (58.1)	400 (50.1)	
Female	804 (45.5)	406 (41.9)	398 (49.9)	
Race				<.05
Chicano	329 (18.7)	181 (18.7)	148 (18.5)	
Other Hispanic	188 (10.7)	97 (10.0)	91 (11.4)	
Non-Hispanic White	631 (35.7)	298 (30.7)	333 (41.8)	
Non-Hispanic Black	440 (24.8)	310 (31.9)	130 (16.2)	
Asian American	180 (10.1)	84 (8.7)	96 (12.1)	
Family income				.11
<10,000	32 (3.3)	18 (3.5)	14 (3.2)	
10,000–20,000	73 (7.7)	36 (7.0)	37 (8.5)	
20,000–50,000	163 (17.3)	80 (15.7)	83 (19.2)	
50,000–75,000	131 (13.9)	63 (12.5)	68 (15.7)	
75,000–100,000	159 (16.8)	90 (17.7)	69 (15.9)	
Above 100,000	209 (22.1)	119 (23.3)	90 (20.8)	
Not informed	175 (18.9)	104 (20.3)	71 (16.7)	
Smoking				.06
Yes	265 (30.8)	157 (33.5)	108 (27.6)	
No	593 (69.2)	311 (66.5)	282 (72.4)	
Drinking				.04
Yes	794 (62.9)	466 (65.4)	328 (59.8)	
No	468 (37.1)	247 (34.6)	221 (40.2)	
Hypertension				<.05
Yes	785 (45.0)	232 (25.9)	295 (34.8)	
No	959 (55.0)	664 (74.1)	553 (65.2)	
25-hydroxyvitamin (nmol/L)	60.23 ± 23.64	57.49 ± 22.75	63.82 ± 24.36	.005
BMI/(kg/m^3^)	30.74 ± 5.83	32.28 ± 5.93	29.01 ± 5.33	<.05
Glycosylated hemoglobin %	7.31 ± 1.73	7.55 ± 1.82	7.23 ± 1.52	<.05
Waistline/cm	106.12 ± 14.15	109.17 ± 14.03	103.34 ± 13.65	<.05
Calcium serum (mmol/L)	2.36 ± .10	2.36 ± .09	2.36 ± 0.10	.626
Serum phosphorus (mmol/L)	1.21 ± .18	1.21 ± .19	1.21 ± 0.18	.512
Lithic acid (μmol/L)	336.42 ± 90.65	336.76 ± 90.76	335.99 ± 90.61	.882
Fasting blood-glucose (mmol/L)	8.81 ± 3.80	9.19 ± 3.91	8.33 ± 3.62	.004

BMD = bone mineral density, BMI = body mass index.

### 3.2. Association between DII and low BMD

The association between DII score and low BMD was examined using binary logistic regression (Table 2). Among women, a higher DII was significantly associated with a greater risk for low BMD in both the femoral neck and lumbar spine (femoral neck: model 3 odds ratio [OR] = 1.087, 95% confidence interval [CI] = 1.036–1.140, *P* = .001; lumbar spine: model 3 OR = 1.052, 95% CI = 1.006–1.100, *P* = .026). When DII was categorized into tertiles, women in T_3_ had a higher risk than those in T_1_ (femoral neck: model 3 OR = 2.575, 95% CI = 1.484–4.468, *P* = .001; lumbar spine: model 3 OR = 1.864, 95% CI = 1.111–3.129, *P* = .018).

**Table 2:** Logistic regression analysis results.

Female femoral neck BMD	Model 1		Model 2		Model 3	
	OR (95% CI)	*P*	OR (95% CI)	*P*	OR (95% CI)	*P*
Continuous variable						
DII	1.067 (1.019–1.118)	.006	1.075 (1.027–1.126)	.002	1.087 (1.035–1.141)	.001
Classified variable						
T1	Ref	–	Ref	–	Ref	–
T2	1.663 (0.989–2.798)	.055	1.937 (1.142–3.286)	.014	1.968 (1.100–3.519)	.022
T3	2.134 (1.273–3.578)	.004	2.339 (1.385–3.952)	.001	2.598 (1.485–4.545)	.001

Female lumbar spine BMD	Model 1		Model 2		Model 3	
	OR (95% CI)	*P*	OR (95% CI)	*P*	OR (95% CI)	*P*
Continuous variable						
DII	1.053 (1.006–1.102)	.027	1.052 (1.005–1.102)	.03	1.053 (1.007–1.102)	.022
Categorical variable						
T1	Ref	–	Ref	–	Ref	–
T2	2.029 (1.200–3.431)	.008	2.153 (1.247–3.717)	.006	2.329 (1.293–4.194)	.005
T3	1.872 (1.118–3.135)	.017	1.881 (1.113–3.179)	.018	1.889 (1.119–3.188)	.017

Male femoral neck BMD	Model 1		Model 2		Model 3	
	OR (95% CI)	*P*	OR (95% CI)	*P*	OR (95% CI)	*P*
Continuous variable						
DII	1.006 (0.975–1.039)	.705	0.989 (0.955–1.025)	.556	0.990 (0.951–1.030)	.638
Categorical variable						
T1	Ref	–	Ref	–	Ref	–
T2	0.908 (0.584–1.413)	.67	0.747 (0.474–1.179)	.211	0.868 (0.529–1.424)	.578
T3	1.312 (0.814–2.115)	.265	1.114 (0.651–1.906)	.695	1.186 (0.651–2.160)	.576

Male lumbar spine BMD	Model 1		Model 2		Model 3	
	OR (95% CI)	*P*	OR (95% CI)	*P*	OR (95% CI)	*P*
Continuous variable						
DII	0.967 (0.935–1.000)	.052	0.964 (0.932–0.998)	.038	0.955 (0.919–0.993)	.021
Categorical variable						
T1	Ref	–	Ref	–	Ref	–
T2	0.659 (0.409–1.060)	.086	0.636 (0.397–1.020)	.060	0.663 (0.406–1.082)	.101
T3	0.887 (0.534–1.473)	.643	0.864 (0.515–1.448)	.579	0.767 (0.430–1.367)	.369

Model 1 is the unadjusted model; Model 2 adjusted for race and age; Model 3, additionally adjusted for family income, waist circumference, body mass index, glycated hemoglobin, and smoking status. “Ref” indicates the reference category; “–” indicates not applicable.

BMD = bone mineral density, CI = confidence interval, DII = Dietary Inflammatory Index, OR = odds ratio.

Among men, no significant association was observed for the femoral neck when the DII was modeled continuously (model 3: OR = 0.993, 95% CI = 0.957–1.032, *P* = .733), and tertile comparisons were similarly null (T_2_ vs T_1_: OR = 0.835, 95% CI = 0.512–1.360, *P* = .469; T_3_ vs T_1_: OR = 1.203, 95% CI = 0.672–2.157, *P* = .534). For the lumbar spine, a modest inverse association emerged for continuous DII (model 1 [borderline], OR = 0.967, *P* = .052; model 2 [significant], OR = 0.964, *P* = .038; model 3 [significant], OR = 0.959, 95% CI = 0.924–0.996, *P* = .03), whereas the tertile analyses were not statistically significant (T_2_ vs T_1_: OR = 0.636, 95% CI = 0.391–1.036, *P* = .069; T_3_ vs T_1_: OR = 0.776, 95% CI = 0.446–1.351, *P* = .37).

Overall, associations between DII and low BMD differed according to sex: positive associations were consistently observed in women, whereas no positive association was observed in men, and the inverse association for the lumbar spine in men was modest and not supported by tertile analyses and, thus, should be interpreted cautiously.

**Figure 2.**
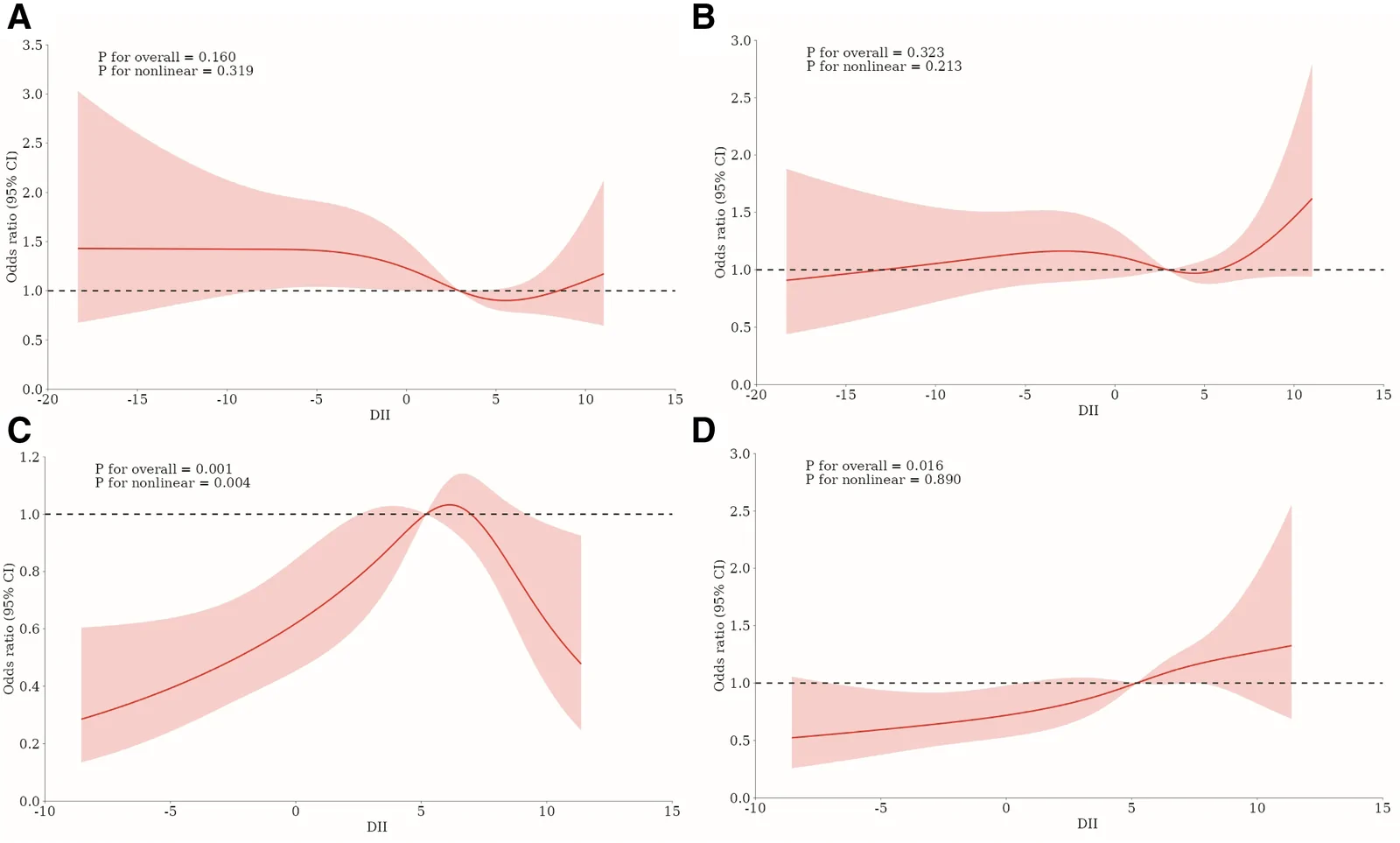
Sex-stratified restricted cubic spline curves for the association between the DII and low BMD. (A) Lumbar spine in men; (B) femoral neck in men; (C) lumbar spine in women; and (D) femoral neck in women. The horizontal axis represents the DII score, and the vertical axis represents the adjusted OR for low BMD. Solid red lines represent the fitted restricted cubic spline curves, and shaded areas represent the corresponding 95% CIs. The sex-specific median DII score was used as the reference value, with an odds ratio of 1. Models were adjusted for age, race/ethnicity, household income, waist circumference, body mass index, glycated hemoglobin, and smoking status. *P* for overall indicates the overall association, and *P* for nonlinearity indicates departure from linearity. BMD = bone mineral density, CI = confidence interval, DII = Dietary Inflammatory Index, OR = odds ratio.

Restricted cubic spline analyses revealed evidence of a nonlinear association between the DII and lumbar spine outcomes in women (Fig. 2). The corresponding spline curves and nonlinear test results are presented in Figure 2.

**Figure 3.**
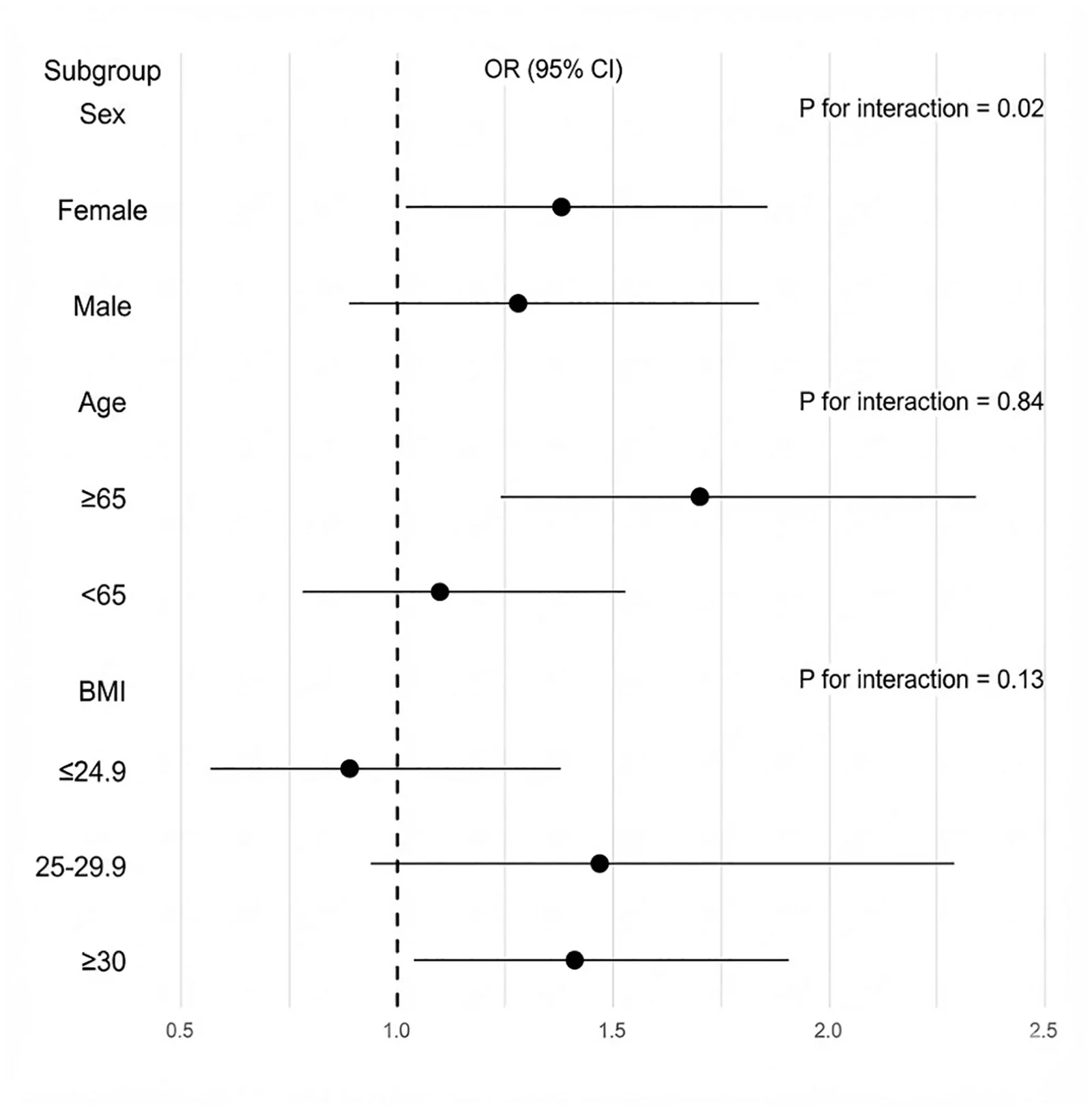
Subgroup analyses of the association between the DII and low BMD among adults with type 2 diabetes. Results are shown by sex, age, and BMI. Circles indicate ORs, horizontal lines indicate 95% CIs, and the vertical dashed line indicates an OR of 1. *P* values for interaction assess differences across subgroups. BMD = bone mineral density, BMI = body mass index, CI = confidence interval, DII = Dietary Inflammatory Index, OR = odds ratio.

Stratified analyses (Fig. 3) revealed a significant interaction according to sex in the association between DII scores and risk for low BMD among patients with diabetes (*P* for interaction = .02), indicating that the effect of DII may differ between women and men. In contrast, the interactions with age (*P* for interaction = .84) and BMI (*P* for interaction = .13) were not significant, suggesting that the DII–OP relationship was consistent across the age and BMI strata.

**Figure 4.**
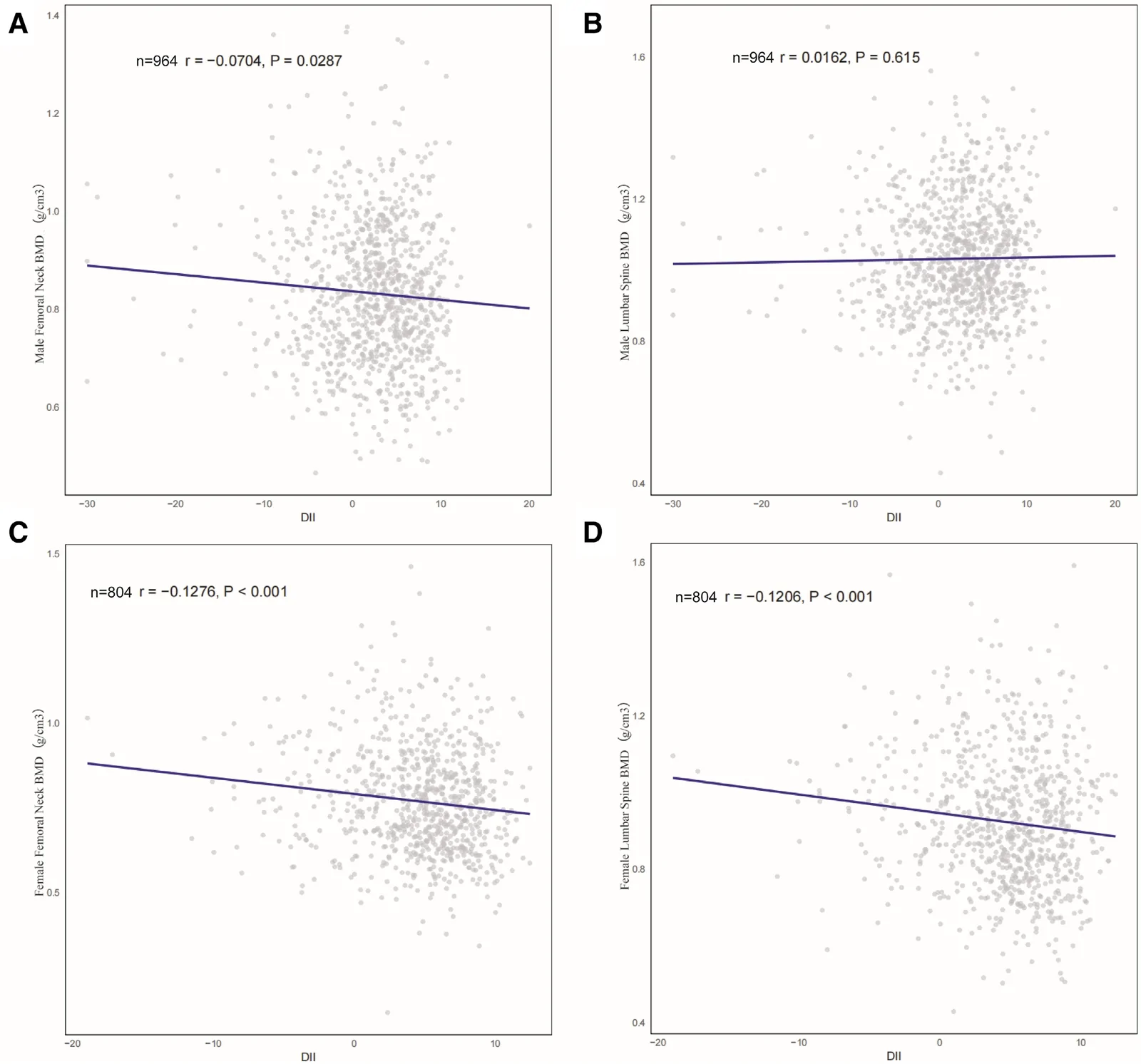
Sex-stratified scatterplots showing the correlations between the DII and BMD at the femoral neck and lumbar spine. (A) Femoral neck BMD in men; (B) lumbar spine BMD in men; (C) femoral neck BMD in women; and (D) lumbar spine BMD in women. The horizontal axis represents the DII score, and the vertical axis represents BMD in g/cm^2^. Each point represents 1 participant, and the solid line represents the fitted linear regression line. Pearson correlation coefficients (*r*), sample sizes, and corresponding two-sided *P* values are displayed in each panel. BMD = bone mineral density, DII = Dietary Inflammatory Index.

Correlation analyses between DII and BMD revealed significant negative associations for the femoral neck in men (*r* = −0.0704, *P* = .028) and for both the femoral neck (*r* = −0.127, *P* < .001) and lumbar spine (*r* = −0.1206, *P* < .001) in women. In contrast, no significant correlation was observed between the DII and lumbar spine BMD in men (*r* = 0.0162, *P* = .615). These findings suggest that higher DII scores adversely affect BMD in women (at both the femoral neck and lumbar spine), whereas in men, the effect appears to be confined to the femoral neck (Fig. 4).

## 4. Discussion

Using NHANES data, we observed sex-specific associations between the DII and bone outcomes among adults with diabetes. Higher DII scores were associated with higher odds for low BMD (osteopenia/OP) in women and were inversely correlated with BMD in both the femoral neck and lumbar spine. Among men, the associations were generally weak and were not statistically significant in most analyses. Collectively, these findings are consistent with the concept that chronic low-grade inflammation may contribute to diabetes-related bone impairment and suggest that dietary inflammatory potential may have differential relevance according to sex. Epidemiological evidence also supports sex- and site-specific heterogeneity in the DII–bone relationship. In an NHANES 2017 to 2018 analysis of adults ≥50 years of age, higher DII was associated with lower total hip and femoral neck BMD and higher odds for OP in women, whereas in men, DII was associated with lower lumbar spine BMD without a corresponding increase in OP risk.^[19]^ Consistently, prospective data from the Women’s Health Initiative showed that a more pro-inflammatory diet was associated with lower BMD and a higher risk for fractures among postmenopausal women.^[20]^ Among men, the inverse association observed for lumbar spine outcomes when the DII was modeled continuously was small and was not corroborated by tertile-based analyses. Therefore, this finding should be considered weak, exploratory, and may reflect residual confounding or measurement-related factors; nevertheless, our main conclusions are concentrated in women.

Previous studies have linked higher DII scores to chronic inflammation-related conditions, including cardiovascular disease, cognitive impairment, and cancer.^[21]^ Extending this literature to bone health, our results indicated that a more pro-inflammatory dietary profile is associated with worse bone outcomes in women with diabetes. Several mechanisms have been proposed in previous research, and we interpret the following pathways to be biologically plausible explanations rather than direct evidence from the present cross-sectional analysis.

First, evidence from observational studies suggests that more anti-inflammatory dietary patterns are associated with slower bone loss, with particularly notable associations reported in postmenopausal women and individuals with diabetes.^[22,23]^ A decline in estrogen levels after menopause may reduce the suppression of inflammatory signaling, including the NF-κB pathway.^[24]^ A diet reflecting higher DII scores may further amplify inflammatory activity, as reflected by higher levels of inflammatory biomarkers such as IL-6 and CRP.^[25]^ In addition, experimental evidence indicates that pro-inflammatory states can increase oxidative stress and may inhibit osteogenic pathways such as Wnt/β-catenin signaling through reactive oxygen species, thereby impairing osteoblast mineralization.^[26]^ Oxidative stress–linked mechanisms such as ferroptosis have also been implicated in osteoblast dysfunction; for example, baicalein was reported to alleviate iron overload–induced osteoblast ferroptosis and osteogenic blockade by activating the Nrf2/GPX4 pathway, supporting a plausible oxidative stress–bone axis relevant to OP biology.^[27]^ Collectively, these data support a plausible link between diet-related inflammation and compromised bone remodeling, which may be more pronounced in women.

Second, sex differences in skeletal composition and the endocrine milieu may contribute to heterogeneous susceptibility. Women generally have a higher proportion of trabecular bone, which is metabolically active and may be more responsive to inflammatory perturbations.^[28]^ In contrast, testosterone-related effects on bone formation may provide a partial compensatory capacity in men.^[29]^ Sociobehavioral factors may also play a role; for example, dietary patterns differing according to sex could influence DII distributions and downstream inflammatory profiles.^[30]^

Results of the present study revealed that the DII was significantly correlated with the lumbar spine and femoral neck BMD in women, whereas only a weak association was observed at the femoral neck in men. This discrepancy may be attributed to the biological differences between skeletal sites. The femoral neck, which is rich in cancellous bone, undergoes higher bone turnover rates than cortical bone,^[31]^ making it more sensitive to fluctuations in inflammatory markers. In contrast, lumbar spine BMD measurements are susceptible to interference from non-metabolic factors, such as vertebral degeneration and disc calcification, which occur more frequently in elderly males^[32]^ and, as such, may obscure the actual effect of DII. Furthermore, stratified analyses indicated that the association between DII score and OP risk remained consistent across different age and BMI groups, suggesting that inflammation exerts a universal influence on bone metabolism independent of traditional risk factors.

These findings open new avenues for the prevention and management of diabetes-related OP. For women, greater attention should be devoted to personal dietary habits with timely and appropriate interventions. In particular, postmenopausal women with diabetes are advised to adopt a low-DII diet because research indicates that increasing daily dietary fiber intake by 10 g can significantly reduce CRP levels.^[33]^ Additionally, dynamic monitoring of DII is necessary in high-risk populations. It is recommended that the DII be incorporated into diabetes management guidelines, and regular bone density assessments should be performed, especially in patients with HbA1c levels exceeding 7.5%.

Several limitations of the present study merit emphasis. First, its cross-sectional design precludes establishing temporality or directionality between the DII score and bone outcomes, and reverse causation cannot be excluded. Second, the DII was derived from 24-hour dietary recall, which is subject to recall bias and day-to-day variability, potentially leading to exposure misclassification.^[34]^ Third, residual confounding is possible because not all determinants of diet quality, inflammation, and bone metabolism were fully measured in NHANES. Specifically, individual classes of glucose-lowering medications were not included as covariates in the present analysis. Although NHANES records the names and self-reported duration of prescription medications used during the preceding 30 days, the publicly available data do not consistently capture medication dose, adherence, or detailed treatment changes. Consequently, cumulative exposure to thiazolidinediones could not be reliably characterized, and residual confounding related to their potential effects on bone metabolism and BMD, particularly among women, cannot be excluded. Finally, the lack of hormone measurements (e.g., estrogen and testosterone) limits the mechanistic exploration of sex-specific pathways. Future longitudinal cohort and interventional studies are needed to validate these associations and to clarify the underlying mechanisms.

In summary, a higher DII score was independently associated with OP/low BMD in women with diabetes and demonstrated notable sex- and site-specific patterns. These findings suggest that sex-specific nutritional strategies targeting dietary inflammatory potential may be relevant in diabetes-related bone health and that the DII could be considered a candidate marker for OP/low BMD risk stratification, pending further validation. Future longitudinal and interventional studies, potentially integrating multi-omics technologies, are warranted to elucidate the molecular pathways linking dietary inflammation, oxidative stress, and sex hormones, and to advance precision approaches to treating metabolic bone diseases.

## Author contributions

**Visualization:** Menglin Xu.

**Conceptualization:** Shaocong Li.

**Data curation:** Shaocong Li.

**Software:** Shaocong Li, Qiqi Tang.

**Investigation:** Qiqi Tang.

**Writing – original draft:** Menglin Xu, Shaocong Li.
